# Predictors of mortality and validation of burn mortality prognostic scores in a Malaysian burns intensive care unit

**DOI:** 10.1186/s12873-019-0284-8

**Published:** 2019-11-07

**Authors:** Henry Tan Chor Lip, Mohamad Azim Md. Idris, Farrah-Hani Imran, Tuan Nur’ Azmah, Tan Jih Huei, Mathew Thomas

**Affiliations:** 1 Department of General Surgery, Hospital Sultanah Aminah, Johor Bahru, Malaysia; 2 Department of Surgery, Pusat Perubatan Universiti Kebangsaan Malaysia, Kuala Lumpur, Malaysia; 3 Department of General Surgery, Hospital Sultan Ismail, Johor Bahru, Malaysia

**Keywords:** Burn, Prognostic score, Mechanical ventilation, Burn scores, Mortality

## Abstract

**Background:**

Majority burn mortality prognostic scores were developed and validated in western populations. The primary objective of this study was to evaluate and identify possible risk factors which may be used to predict burns mortality in a local Malaysian burns intensive care unit. The secondary objective was to validate the five well known burn prognostic scores (Baux score, Abbreviated Burn Severity Index (ABSI) score, Ryan score, Belgium Outcome Burn Injury (BOBI) score and revised Baux score) to predict burn mortality prediction.

**Methods:**

Patients that were treated at the Hospital Sultan Ismail’s Burns Intensive Care (BICU) unit for acute burn injuries between 1 January 2010 to 31 December 2017 were included. Risk factors to predict in-patient burn mortality were gender, age, mechanism of injury, total body surface area burn (TBSA), inhalational injury, mechanical ventilation, presence of tracheotomy, time from of burn injury to BICU admission and initial centre of first emergency treatment was administered. These variables were analysed using univariate and multivariate analysis for the outcomes of death. All patients were scored retrospectively using the five-burn mortality prognostic scores. Predictive ability for burn mortality was analysed using the area under receiver operating curve (AUROC).

**Results:**

A total of 525 patients (372 males and 153 females) with mean age of 34.5 ± 14.6 years were included. There were 463 survivors and 62 deaths (11.8% mortality rate). The outcome of the primary objective showed that amongst the burn mortality risk factors that remained after multivariate analysis were older age (*p* = 0.004), wider TBSA burn (*p* < 0.001) and presence of mechanical ventilation (p < 0.001). Outcome of secondary objective showed good AUROC value for the prediction of burn death for all five burn prediction scores (Baux score; AUROC:0.9, ABSI score; AUROC:0.92, Ryan score; AUROC:0.87, BOBI score; AUROC:0.91 and revised Baux score; AUROC:0.94). The revised Baux score had the best AUROC value of 0.94 to predict burns mortality.

**Conclusion:**

Current study evaluated and identified older age, total body surface area burns, and mechanical ventilation as significant predictors of burn mortality. In addition, the revised Baux score was the most accurate burn mortality risk score to predict mortality in a Malaysian burn’s population.

## Highlights

- Data is from Malaysia, Southeast Asia nation which found that older age, wider TBSA burn, longer length of stay and mechanical ventilation were significant predictors of burn mortality.
- Mechanical ventilation is a more significant predictor of death compared to inhalational injury in our local burn injured patients
- There was no statistical significance in death and TBSA burn calculation for patients treated at the parent emergency department BICU and patients treated outside. However, there was a significantly longer time to from injury to admission to BICU.
- Amongst the five burn mortality prognostic scores (Baux, ABSI, BOBI, Ryan and Revised Baux), the Revised Baux score had the highest the best predictive ability which may be used in the emergency setting.
- The revised Baux score is simple and can be used as triaging tool on admission for mortality risk counselling.

## Background

Burn injuries represent a significant economic health care burden leading to an estimated 265,000 deaths worldwide per year. Mortality rates remains high (10–20%) even in well-equipped burn centres [1]. According to the World Health Organization (WHO), majority of burn fatalities originated from developing nations in Southeast Asia region with a prevalence of 1.3 burn patients in every 100,000 populations [2]. This data is supported by the Malaysian Ministry of Health 2016 fact sheet, with the outcome of traumatic burn injuries being the 5th highest leading cause of hospitalisation within the Ministry of Health [3]. In Malaysia, the availability of beds in burn intensive care units (BICU) is limited due to the high cost and manpower of maintenance [4]. In light of this situation, burn mortality predictors and prognostic scores is needed to triage severely burned patients in accordance to severity. Majority of such burn prognostic scores (Baux score, Abbreviated Burn Severity Index (ABSI) score, Ryan score, Belgium Outcome Burn Injury (BOBI) score and revised Baux score) were devised on western burn populations which lacks validation from an Asian burn’s population [4].

### Study objective

The primary objective was to evaluate and identify risk factors which may predict outcome of in-patient mortality in a Malaysian burn’s population.

The secondary objective of this study was to validate the five well known burn mortality prognostic score (Baux score, Abbreviated Burn Severity Index (ABSI) score, Ryan score, Belgium Outcome Burn Injury (BOBI) score and revised Baux score) in predicting mortality within a Malaysian burns intensive care unit.

## Methods and materials

This study is a retrospective analysis of the burn database of patients admitted to the burns intensive care unit (BICU) of Hospital Sultan Ismail, Malaysia from 1 January 2010 to 31 December 2017. This hospital’s BICU is the reference centre for all adult burn patients for the southern part of peninsular Malaysia. The hospital’s BICU is a subspecialty of general surgery which receives referrals from the emergency department, local district clinics, hospitals and inter-departmental referrals for burn injuries within southern Malaysia.

The BICU is managed by a burn and trauma surgeon, anaesthetists, a medical officer and 17 nurses trained in managing burn wounds. Patient details from admission to discharge is recorded within the hospital’s electronic burns registry. Any discrepancies in data is discussed with the burn’s surgeon first, prior to data entry ensuring accuracy and prevent missing data. Data collection began upon admission, recorded prospectively throughout the hospital admission and completed on discharge or death.

### Patient population

Prior to data collection, ethics approval was granted by the Medical Research and Ethics Committee of Malaysia, KKM.NIHSEC/P17–1904(5) in accordance with current guidelines on Good Clinical Practice, the Declaration of Helsinki, and subsequent relevant versions. The inclusion criteria were all burn patients aged ≥13-years-old, patients with major burns, and all patients with burns involving the face, hands, feet, genitalia, perineum or major joints, electrical and chemical burns. The exclusion criteria were patients that were transferred in from other BICUs for continuation of burn wound care and patients with missing data.

In our centre, major burns were defined as all partial and full-thickness burns with total burn surface area (TBSA) > 20% involving burns on the face, hands feet, genitalia, perineum or major joints, inhalational injury, electrical and chemical burns. TBSA estimation is performed using the Lund and Browder chart. Inhalational injury is defined as all burn patients with a history of being in an enclosed burn space and clinical features which may include a singed eye brow, soot in nostrils, laryngeal oedema and facial burns that may suggest possible inhalational injury. Indication for mechanical ventilation for patients with burn inhalational injury is based on clinical and blood parameters coupled with the clinical experience of the attending anaesthetist. Positive clinical findings of rales, ronchi, wheezing, tachypnea and suspicious visible supraglottic oedema are main factors for mechanical ventilation. Subsequent evidence of hypoxia by the arterial blood gas results further lowers the threshold for mechanical ventilation. All severely burn patients were managed and resuscitated initially according to the advanced trauma life support (ATLS) and American Burn Association (ABA) guidelines. All patients with over 20% partial to full thickness burns received the Parkland Formula for fluid resuscitation. Patients with inhalational injury received treatment in accordance to the hospital’s inhalational injury treatment protocol. The protocol includes humidified oxygen to maintain oxygen saturation above 90%, chest physiotherapy, two hourly turn and positioning, aerosolised N-acetylcysteine with bronchodilator, alternate aerosol 5000 units of Heparin together with normal saline, regular airway suction and sputum cultures. Nebulised Heparin was given to inhibit pulmonary fibrin clot formation and improve oxygenation for patients with inhalational injury.

### Study variables and burn mortality prognostic scores

Variables chosen to predict mortality during the hospital stay included gender, age, mechanism of injury, total body surface area burn, inhalational injury, mechanical ventilation, tracheotomy, length of time from injury to BICU admission and centre of initial emergency treatment was administered prior transfer/admission to BICU. Centre of initial emergency treatment were categorized into the hospital’s emergency department (ED BICU) and outside of the parent emergency department categorized as Periphery Primary and Secondary Health Care Centre (PPSHCC).

The risk of death from burn injury for each patient was estimated using five burn scores: the Baux score, Abbreviated Burn Severity Index (ABSI), Ryan score, Belgium Outcome Burn Injury (BOBI) and revised Baux score. These scores were calculated based on the recorded variables in the burns database. The Baux score is the summation of patient’s age and TBSA, equivalent to the percentage of mortality [5]. The ABSI is a scoring system based on the parameters of patient’s sex, category of age, presence of inhalational injury and TBSA which is numerically scored between 0 to 17. Higher scores denote a lower probability of survival [6]. The Ryan score uses 3 risk factors of age greater than 60 years, TBSA of 40% or greater and inhalational injury with a maximum score of 1 for each risk factor present. Higher score represents a higher risk of mortality for death [7]. The BOBI score uses categorical values of age, TBSA and presence of inhalational injury [8]. The maximum score is 10 which give a 99% risk of mortality. Lastly, the revised Baux score includes the presence of inhalational injury giving the percentage of mortality [5]. The details of these five scores are seen in Additional file 1.

### Statistical analysis

All analysis of data was performed using SPSS for Windows version 16.0 (SPSS Inc., Chicago, USA). All continuous variables were expressed as mean and standard deviation (SD) and categorical variables were expressed as frequencies and percentages. For each variable/risk factor of interest, univariate analysis performed between survivors and non-survivors predicting death with significance accepted *p* < 0.05. The univariate analysis was performed using Chi square test, Student’s t test or Mann-Whitney test where appropriate. All variables with significant *p* value by univariate analysis were included in the multivariate binary logistic regression using enter method to determine association with mortality.

The five burn mortality prediction scores (Baux score, Abbreviated Burn Severity Index (ABSI) score, Ryan score, Belgium Outcome Burn Injury (BOBI) score and revised Baux score) were evaluated using area under receiver operating curve (AUROC) to determine the accuracy at distinguishing between survivors (false positive) and non-survivors (true positives). An area over 0.9 indicates high accuracy, 0.7–0.9 moderate accuracy 0.5–0.7 low accuracy and 0.5 indicates chance discrimination [9].

## Results

A total of 525 patients were treated for burn injuries at the BICU of Hospital Sultan Ismail, Malaysia, 372 males and 153 females, all of whom fulfilled the inclusion and exclusion criteria. The mean ages of patients were from the active working-class population of 34.5 ± 14.6 years. The mean length of BICU stay was 17.3 ± 21.2 days. The average percentage of total body surface area burn (cumulative of second- and third-degree burns) was 19.8 ± 19.9%. One hundred and fifty-four patients had inhalational injury, in which 141 patients required mechanical ventilation (Table 1). There were 62 (11.8%) mortalities and 463 (98.2%) survivors. These groups were categorised into the survivor and non-survivor group and its clinical characteristics is seen in Table 2. Majority of the non-survivors were males with a mean age of 39.85 ± 16.82 years. Non-survivors had a greater mean TBSA (50 ± 26.08%), had inhalational injuries (50/62 patients; 80.7%) and ventilated mechanically (59/62 patients; 95.2%). Sub-analysis comparing patients that received initial emergency treatment at the ED BICU, or PPSHCC, is listed in Table 3.

**Table 1:** Clinical characteristics of all patients treated in burns intensive care unit

Variable	All Cases (*n* = 525)
Sex	
male	372 (70.9%)
female	153 (29.1%)
Mean Age (years)*	34.5 ± 14.6
Age group (years)	
≤ 18	46 (8.8%)
19–49	394 (75.0%)
50–64	60 (11.4%)
65–74	18 (3.4%)
≥ 75	7 (1.3%)
Race	
Malay	247 (47.0%)
Chinese	104 (19.8%)
Indian	45 (8.0%)
Others	132 (25.1%)
Place of injury	
Household	285 (54.3%)
Industrial/workplace	167 (31.8%)
Road traffic accident	28 (5.3%)
Others	45 (8.6%)
Mechanism of injury	
Thermal	450 (85.7%)
Chemical	51 (9.7%)
Electrical	17 (3.2%)
TBSA burn *	19.8 ± 19.9%
Inhalational Injury	154 (29.3%)
Mechanical ventilation	141 (26.9%)
Tracheostomy	24 (4.6%)

*mean (SD); n, number; TBSA, total body surface area

**Table 2:** Univariate analysis and comparison of clinical characteristics of survivors and mortality

Variable	Survivor (*n* = 463)/%	Non-survivor (*n* = 62)/%	*p*-value	95% CI
Sex (male/female) ♂				
Male	325 (70.2%)	47 (75.8%)	0.361	0.72–2.459
Female	138 (29.8%)	15 (24.2%)		
Age (years)*♀	33.7 ± 14.2	39.9 ± 16.8	0.001	–
Place of injury: ♂				
Household	256 (55.3%)	29 (46.8%)	0.132	
Industrial/workplace	148 (32%)	19 (30.6%)		–
Road traffic accident	24 (5.2%)	4 (6.5%)		
Others	35 (7.6%)	10 (16.1%)		
Mechanism of injury: ♂				
Thermal	398 (86%)	52 (83.9%)	0.969	
Chemical	44 (9.5%)	7 (11.3%)		–
Electrical	15 (3.2%)	2 (3.2%)		
TBSA burn*♀	15.7 ± 13.2%	50.5 ± 26.1%	0.021	–
Inhalational Injury♂	104 (67.5%)	50 (80.7%)	< 0.001	7.38–28.0
Mechanical ventilation♂	82 (22.5%)	59 (95.2%)	< 0.001	27.9–298.7
Tracheostomy♂	13 (2.8%)	11 (17.7%)	< 0.001	3.18–17.5

*mean (SD); ♂, Chi-Square test, ♀, t-test; *n, number; LOS, length of stay; TBSA, total body surface area.*

**Table 3:** Comparison of patients treated at district hospital first and patients treated at emergency department of BICU

Variable	ED BICU (*n* = 261)	PPSHCC (*n* = 264)	p-value
Time from injury to BICU admission (hours)*	2.37 ± 1.65	9.5 ± 17.38	< 0.001
First assessment percentage of TBSA burn*	20.25 ± 19.61%	21.16 ± 18.84%	0.379
Difference of estimated percentage TBSA from BICU*	1.2 ± 5.19%	0.61 ± 5.16%	0.188
Total deaths	26 (41.94%)	36 (58.06%)	0.192

*mean (SD); n, number, TBSA percentage total body surface area, ED, Emergency Department, BICU Burns Intensive Care Unit, PPSHCC Periphery Primary and Secondary Health Care Centre

From the initial univariate analysis, significant independent of age, total body surface area burned, mechanical ventilation, tracheostomy and inhalational injury were included for multivariate analysis (Tables 2 and 3). When these variables were combined for multivariate analysis, the significant independent variables that remained were age, total body surface area burned and mechanical ventilation. The following odds ratio is presented in Table 4.

**Table 4:** Multivariate analysis for factors influencing in hospital death

Variable	P-Value	OR	95% CI
Age (years)*	0.004	1.04	1.01–1.07
TBSA burn*	< 0.001	1.06	1.04–1.08
Mechanical ventilation	< 0.001	32.49	7.04–150.03
Tracheostomy	0.946	0.96	0.33–2.85
Inhalational Injury	0.984	0.99	0.32–3.05

*mean (SD); TBSA, percentage total body surface area

**Fig. 1:**
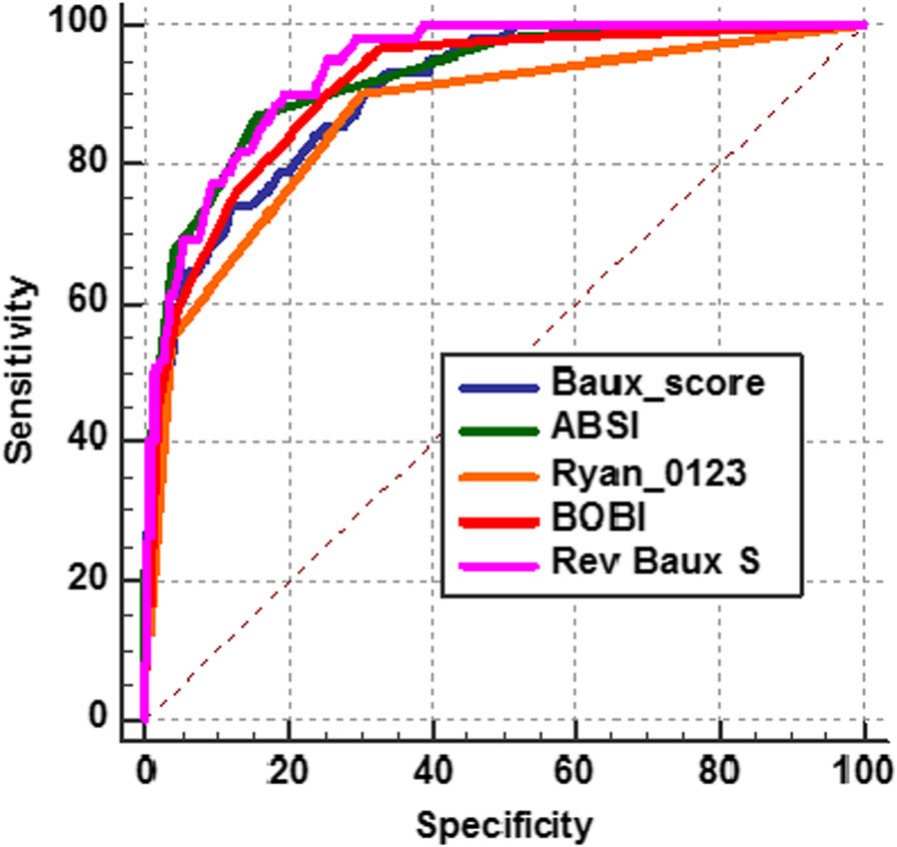
AUC curve for Baux, ABSI, Ryan, BOBI and Revised Baux score

The mean burns mortality scores comparing the Baux, ABSI, Ryan, BOBI and Revised Baux score is seen in Table 3. The mean scores were Baux (54 ± 24.18), ABSI (5.41 ± 2.51), Ryan (2.48 ± 0.7), BOBI 1.75 ± 2.06) and Revised Baux score (59.08 ± 27.66). The mean scores were higher in the non-survivor group which predicts death from the severity of burn injuries. AUROC curve and its values were used to determine the predictive ability of each score to determine burn death in our burn centre Table 5. The AUROC value for each score had a good predictive with the revised Baux score with the highest AUROC value of 0.94 followed by the ABSI score of 0.92. The lowest AUROC score was produced from the Ryan score with AUROC value of 0.867. The individual AUROC graphs can be seen in Fig. 1. No further analysis was performed in view of all the burn scores had good AUROC values to predict burn mortality.

**Table 5:** Comparison of burn mortality mean scores and AUC values

Burn Mortality	All Cases	Survivor	Death	Sensitivity	Specificity	AUC (95%CI)
Scores	(n = 525)	(n = 463)	(n = 62)			
Baux	54 ± 24.18	49.12 ± 19.48	90.43 ± 24.87	74.2	88.1	0.9 (0.88–0.93)
ABSI	5.41 ± 2.51	4.83 ± 1.83	9.68 ± 2.76	87.1	84.2	0.92 (0.9–0.95)
Ryan	0.48 ± 0.7	0.34 ± 0.56	1.52 ± 0.76	90.3	70	0.87 (0.84–0.89)
BOBI	1.75 ± 2.06	1.32 ± 1.67	4.95 ± 1.88	90.3	74.7	0.91 (0.88–0.94)
Revised Baux	59.08 ± 27.66	53.02 ± 21.63	104.16 ± 26.05	90.3	80.6	0.94 (0.91–0.96)

*n, number; ABSI, Abbreviated Burn Severity Index; BOBI, Belgium Outcome in Burn Injury, AUC; Area under the curve valu*

## Discussion

### Predictors of burn mortality

Older age is often associated with physiological reduction in immune function and thinning of the skin [10]. This is often the reason for longer recovery periods for older patients with burn. In general, the elderly population is at higher risk of mortality due to increased pre-existing medical co-morbidities, failure of immune system to combat post-burn infections and thinning of skin which leads to deeper burn injuries [11]. Although the acceptable definitions of elderly age group were usually taken as 65 years and older, there are reports that rebuke that age alone is not a significant predictor. The use of a frailty score may better predict mortality in elderly burn population [12].

Greater total body surface area burn is a well-known predictor of burn mortality. *Jeschke* et al reports that adults with 40% total body surface area burn are at a high risk for mortality and morbidity even in a highly specialised burn centre [12]. The common cause of death is due to reduced immune function due to loss of skin coverage that leads to wound sepsis and hospital complications of pneumonia [11].

Mechanical ventilation is indicated in the majority of patients with inhalational injuries. Although some may succumb to severe lung injury, the majority of patients recover. Complications of ARDS and pneumonia may occur after mechanical ventilation [13]. Although it is not clear whether these complications occur after the primary lung insult or secondary to mechanical ventilation, there is a strong statistical significance seen between mechanical ventilation and mortality seen in this current study.

### Initial admitting facility and time from injury to burn unit admission

Due to the limited BICU centres in Malaysia, majority of burn patients may be inevitably treated at a non-burn centre prior to referral to a BICU. Therefore, this study attempts to explore other associated risk factors of the centre administering emergency initial burn treatment with mortality. The time of burn injury to burn unit admission was longer in patients that were treated first at PPSHCC. Time from burn to admission was longer due to the initial phase of emergency resuscitation, logistic and communications with the burn centre and arranging appropriate transportation (land or air transfers) specific to geographical location. Although it was not statistically significant but the time to admission had positive odds for mortality in the multivariate analysis. *Hrenjec* et al stated that the admitting facility is one of the critical factors predicting mortality in burn patients [14]. This study did not reveal specific centres but identified high volume burn centres had better outcomes in terms of survival.

### Burn mortality prediction scores

Amongst the five burn scores which were validated in this current study, the revised baux score had the highest area under receiver operating curve value of 0.94 to predict burn mortality in our local Malaysian burn population. The result is obvious as it incorporates well known burn mortality predictors of age, total body surface area burns and inhalational injury. The remaining burn scores had good predictive ability which is due to the incorporation of similar burn mortality predictors. From the results, it is advocated to use the revised Baux score in a Malaysian burn unit mainly because of its simplicity of the addition of age, TBSA burn and plus 17 (if there is presence of inhalational injury) which the sum, is equivalent to the percentage of mortality in the individual burn patient. With this validation, it may be crucial for prognostic and mortality risk counselling to patient relative during the initial resuscitative phase [15, 16].

### Study limitation

Despite validating these burn mortality scores, there is a possible higher significant number of deaths in patients which were involved with blunt trauma. This is especially seen in road traffic accident patients which may have polytrauma which may lead to death instead of the burn injury. This is a limitation of this current study which may possibly addressed in future retrospective studies. In addition, although there were dedicated burn trauma nurses for data collection, we did not have a luxury to have a data manager. Most of the data collected were through the concerted of nurses under the direct supervision of the burns and trauma surgeon.

## Conclusion

From this study, significant burn mortality predictors identified were older age, wider TBSA burn and presence of mechanical ventilation. The study also showed that the revised Baux score had the best predictive ability in a Malaysian burn population and its use is advocated for mortality risk counselling to severe burn injured patients. Although there was no statistical significance in patients receiving treatment at ED BICU and PPSHCC, there was a longer time of injury to burn unit admission to patients treated outside first of the parent BICU unit.

## Supplementary information

**Additional file 1.** Details on the Baux score, Abbreviated Burn Severity Index score, Ryan score, Belgium Outcome Burn Injury score and revised Baux score.

## Data Availability

The datasets used and analysed during the current study are available from the corresponding author on reasonable request.

## Abbreviations

ABSI: Abbreviated Burn Severity Index
AUC: Area Under Receiver Operating Curve
BICU: Burns intensive care unit
BOBI: Belgium Outcome Burn Injury
ED BICU: Emergency department of Burns Intensive Care Unit
HSI: Hospital Sultan Ismail
PPSHCC: Periphery Primary and Secondary Health Care Centre
TBSA: Total body surface area
WHO: World Health Organization
