# Supplementary material for: Predictors of mortality and validation of burn mortality prognostic scores in a Malaysian burns intensive care unit
Source: BMC Emerg Med. 2019 Nov 7;19:66. doi: 10.1186/s12873-019-0284-8 (PMC6839226; doi:10.1186/s12873-019-0284-8)
Supplement: Supplementary file 1 — Additional file 1. Details on the Baux score, Abbreviated Burn Severity Index score, Ryan score, Belgium Outcome Burn Injury score and revised Baux score. [file 12873_2019_284_MOESM1_ESM.docx]

Supplement 1: List of five well-known burn mortality risk scoring system.

1. Baux score [6]

Age (years) + Total Body Surface Area Burn (percentage, %) = percentage of mortality

1. Abbreviated Burn Severity Index [7]

| **Abbreviated Burn Severity Index (ABSI)** | |  |
| --- | --- | --- |
| Variable | Patient Characteristics | Score |
| Sex | Female | 1 |
|  | Male | 0 |
| Age in years | 0-20 | 1 |
|  | 21-40 | 2 |
|  | 41-60 | 3 |
|  | 61-80 | 4 |
|  | 81-100 | 5 |
| Inhalation Injury | yes | 1 |
| Full Thickness Burn | yes | 1 |
| Total Body Surface Area burned | 1-10% | 1 |
|  | 11-20% | 2 |
|  | 21-30% | 3 |
|  | 31-40% | 4 |
|  | 41-50% | 5 |
|  | 51-60% | 6 |
|  | 61-70% | 7 |
|  | 71-80% | 8 |
|  | 81-90% | 9 |
|  | 91-100% | 10 |
|  |  |  |
| Total Burn Score | Threat to life | Probability of survival |
| 2 - 3 | Very low | ≥99% |
| 4 - 5 | Moderate | 98% |
| 6 - 7 | Moderately severe | 80-90% |
| 8 - 9 | Serious | 50-70% |
| 10 - 11 | Severe | 20-40% |
| 12 - 13 | Maximum | ≤10% |

1. Ryan Score [8]

| **Ryan Score** |  |
| --- | --- |
| Risk Factor | Point |
| Age > 60 years | 1 |
| Total Body Surface Area Burned >40% | 1 |
| Inhalation Injury | 1 |
|  |  |
| Sum of points | Percentage of mortality |
| 0 | 0.30% |
| 1 | 3% |
| 2 | 33% |
| 3 | 90% |

1. Belgium Outcome in Burn Injury (BOBI) score [9]

| **Belgium Outcome in Burn Injury (BOBI) score** | | | | |  |  |  |  |  |  |  |  |
| --- | --- | --- | --- | --- | --- | --- | --- | --- | --- | --- | --- | --- |
|  | 0 | | 1 | | 2 | | 3 | | 4 | | Score | |
| Age (years) | <50 | | 50-64 | | 65-79 | | ≥80 | |  | | 0-3 | |
| Burned surface area | <20 | | 20-39 | | 40-59 | | 60-70 | | ≥80 | | 0-4 | |
| Inhalation Injury | No | |  | |  | | Yes | |  | | 0-3 | |
| Total |  |  |  |  |  |  |  |  |  |  | 0-10 | |
|  |  |  |  |  |  |  |  |  |  |  |  |  |
|  | Total Score | | | | | | | | | | | |
|  | 0 | 1 | 2 | 3 | 4 | 5 | 6 | 7 | 8 | 9 | 10 |  |
| Predicted mortality (%) | 0 - 1 | 1-5 | 5 | 10 | 20 | 30 | 50 | 75 | 85 | 95 | 99 |  |

1. Revised Baux score [6]

Age (years) + Total Body Surface Area Burn (percentage, %) +17 (if presence of inhalation injury) = percentage of mortality
